# Beta‐catenin participates in dialysate‐induced peritoneal fibrosis *via* enhanced peritoneal cell epithelial‐to‐mesenchymal transition

**DOI:** 10.1002/2211-5463.12182

**Published:** 2017-01-19

**Authors:** Shuiyu Ji, Hao Deng, Wei Jin, Pengpeng Yan, Rending Wang, Lisha Pang, Jingyi Zhou, Jiaming Zhang, Xiaoying Chen, Xiang Zhao, Jia Shen

**Affiliations:** ^1^ Department of NephrologyThe People's Hospital of Zhejiang ProvinceHangzhouChina; ^2^ Kidney Disease CenterThe First Affiliated HospitalZhejiang UniversityHangzhouChina; ^3^ Department of NephrologyThe First People's Hospital of TongxiangChina

**Keywords:** β‐catenin, epithelial‐to‐mesenchymal transition, high glucose, peritoneal dialysis, peritoneal fibrosis

## Abstract

Long‐term exposure to peritoneal dialysate with high glucose (HG) leads to peritoneal fibrosis and thus decreases dialysis efficiency. In this study, we explored the role of β‐catenin in this process. C57BL/6 mice received daily intraperitoneal injection with 10% of the body weight of saline (control), 4.25% glucose peritoneal dialysis fluid (PDF), or PDF combined with 5 mg·kg^−1^ of the β‐catenin inhibitor ICG‐001 (PDF+ICG) for 30 days. Also, mice peritoneal epithelial cells (mPECs) were cultured in 4.25% glucose (HG) or combined with 10 μm ICG‐001 (HG+ICG) for 48 h. We found greater thickness of the parietal peritoneum in the PDF‐treated mice. Additionally, lower expression of E‐cadherin, higher expression of Vimentin, β‐catenin, and Snail, and activation of β‐catenin was observed in the mice and in HG‐treated mPECs, all of which were reversed by ICG‐001. The changes in E‐cadherin and Vimentin indicated occurrence of the epithelial‐to‐mesenchymal transition (EMT). Thus, β‐catenin signaling participates in the process of HG‐induced peritoneal fibrosis, and the EMT of peritoneal epithelial cells is one of the underlying mechanisms of this pathological change.

**Abbreviations**
EMT: epithelial‐to‐mesenchymal transition
HG: high glucose
mPEC: mice peritoneal epithelial cell
PDF: peritoneal dialysis fluid
PD: peritoneal dialysis

Peritoneal dialysis (PD) is an alternative therapy for patients with end‐stage renal disease and is widely used, especially in developing countries. However, long‐term exposure to biologically incompatible PD fluid (PDF) containing high glucose (HG) and its metabolic products tend to interrupt the integrity of the peritoneal membrane, resulting in peritoneal fibrosis and hyperpermeability 1.

The epithelial‐to‐mesenchymal transition (EMT) of peritoneal epithelial cells is considered to be one of the mechanisms underlying peritoneal fibrosis, which starts with the disruption of intercellular junctions, loss of apical‐basolateral polarity, and increased migratory, invasive, and fibrogenic features 2. It has also been demonstrated that myofibroblastic cells may arise from the local conversion of peritoneal mesothelial cells *via* the EMT during PD‐related peritoneal fibrosis 3. The submesothelial and/or subepithelial region, which is composed of connective tissue with fibroblasts, macrophages, mast cells, and vessels, reduces the exchange efficiency and leads to functional decline 4.

Many factors are closely associated with the EMT in patients undergoing PD, such as uremic toxins and the nonphysiological nature of dialysis fluids 5. β‐catenin signaling is associated with the EMT process in renal tubular and alveolar epithelial cells. It also participates in tissue fibrosis in a variety of organs including lung and kidney 6, 7. Nevertheless, its role in peritoneal fibrosis remains unknown.

In this study, we investigated the role of β‐catenin in PDF‐induced peritoneal fibrosis in C57BL/6 mice and HG‐induced fibrotic change in mice peritoneal epithelial cells (mPECs), and its potential relationship with pathological EMT changes.

## Results

### High‐glucose PDF enhances peritoneal fibrosis in C57BL/6 mice

**Figure 1:**
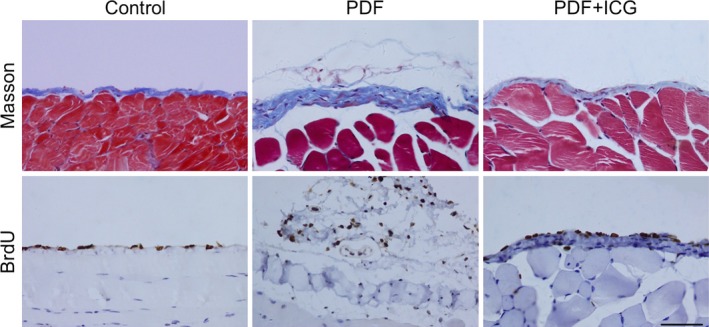
β‐catenin participates in peritoneal fibrosis induced by high‐glucose PDF. Peritoneal tissues were collected 30 days after intraperitoneal injection of 4.25% glucose PDF with or without ICG‐001 (5 mg·kg^−1^·day^−1^). Paraffin sections were stained with Masson's trichrome (blue) or antibody against 5‐bromo‐2‐deoxyuridine (BrdU, brown) (original magnification, ×400; scale bar, 100 μm).

The parietal peritoneum in the control group was thin and covered with a layer of flattened epithelial cells, while long‐term PDF injection resulted in a significantly thicker peritoneum, a loose subepithelial matrix, and collagen accumulation. 5‐Bromo‐2‐deoxyuridine (BrdU) staining also showed higher cell proliferation at the parietal interface in the PDF group than in the control group (Fig. 1).

### High‐glucose PDF increases the EMT in the peritoneal fibrosis process

**Figure 2:**
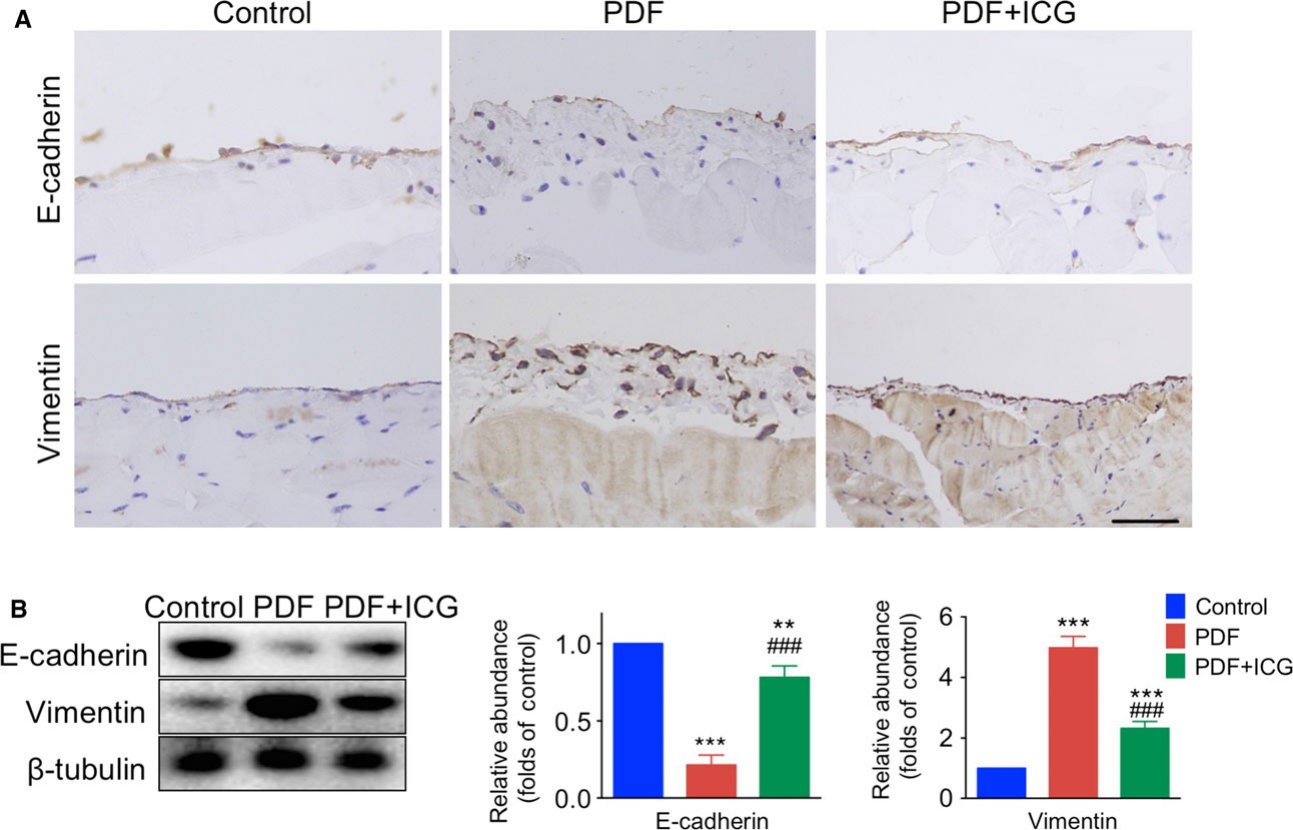
ICG‐001 attenuates the epithelial‐to‐mesenchymal transition in peritoneal fibrosis. (A) Representative images of peritoneal tissues collected 30 days after intraperitoneal injection of 4.25% glucose PDF with or without ICG‐001 (5 mg·kg^−1^·day^−1^). Paraffin sections were stained for E‐cadherin and Vimentin (brown) (original magnification ×400; scale bar, 100 μm). (B) Left panel, western blots of peritoneal tissue lysates with antibodies against E‐cadherin, Vimentin, and β‐tubulin. Right panel, statistics for the expression of E‐cadherin and Vimentin normalized against β‐tubulin (mean ± SD, *n* = 4, **P < 0.01 vs control group, ***P < 0.001 *vs* control group, ^###^ *P* < 0.001 *vs* PDF group).

E‐cadherin is a key epithelial cellular adhesion protein, and Vimentin is an essential component regulating the EMT 8, 9. A loss of E‐cadherin‐positive cells at junctions in the peritoneum and an increased number of Vimentin‐positive cells were found in the PDF group (Fig. 2A). Western blotting showed that E‐cadherin was significantly lower and Vimentin much higher in the PDF group than in the control group (Fig. 2B).

Immunofluorescence showed that while Vimentin‐positive cells were barely seen in mPECs exposed to normal glucose (NG), they were significantly increased in those incubated in HG (Fig. 4A). Western blotting also showed that E‐cadherin expression was much lower and Vimentin expression was much higher in the HG group than in the NG group (Fig. 4B).

### High glucose increases the activation of β‐catenin and Snail expression

**Figure 3:**
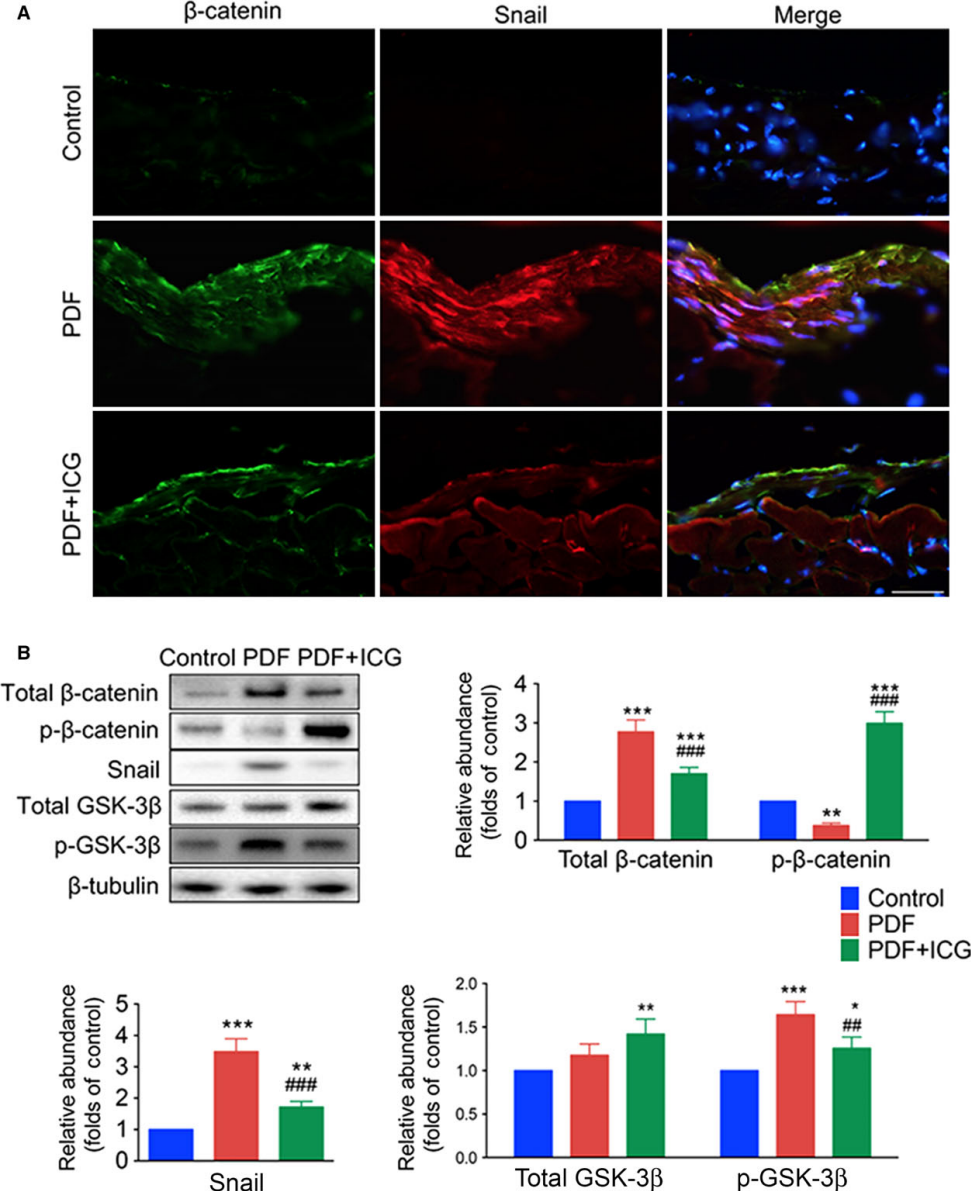
ICG‐001 reactivates GSK‐3β and decreases the expression of Snail and β‐catenin in the peritoneum after PDF exposure. (A) Images of frozen sections stained with antibodies against β‐catenin (green) and Snail (red), nuclei stained with Hoechst (blue) (original magnification, ×400; scale bar, 100 μm). (B) Western blots of peritoneal tissue lysates with antibodies against phosphorylated β‐catenin (p‐β‐catenin), total β‐catenin, Snail, phosphorylated GSK‐3β (p‐GSK‐3β), total GSK‐3β, and β‐tubulin. Statistics of expression levels of p‐β‐catenin, total β‐catenin, Snail, p‐GSK‐3β, and total GSK‐3β normalized against β‐tubulin (mean ± SD, *n* = 4, ***P < 0.001, **P < 0.01, and *P < 0.05 *vs* control group, ^###^ *P* < 0.001 and ^##^ *P* < 0.01 *vs* PDF group).

As transcription generators and EMT markers, up‐regulated cytoplasmic β‐catenin and Snail, both of which are products of β‐catenin target genes, translocate into the nucleus 10. Immunofluorescence demonstrated β‐catenin enrichment and translocation into the nucleus of peritoneal cells after treatment with PDF, while barely any Snail was detectable in the control group. Phosphorylated GSK was up‐regulated by PDF, while total amount of GSK remained steady (Fig. 3A,B).

**Figure 4:**
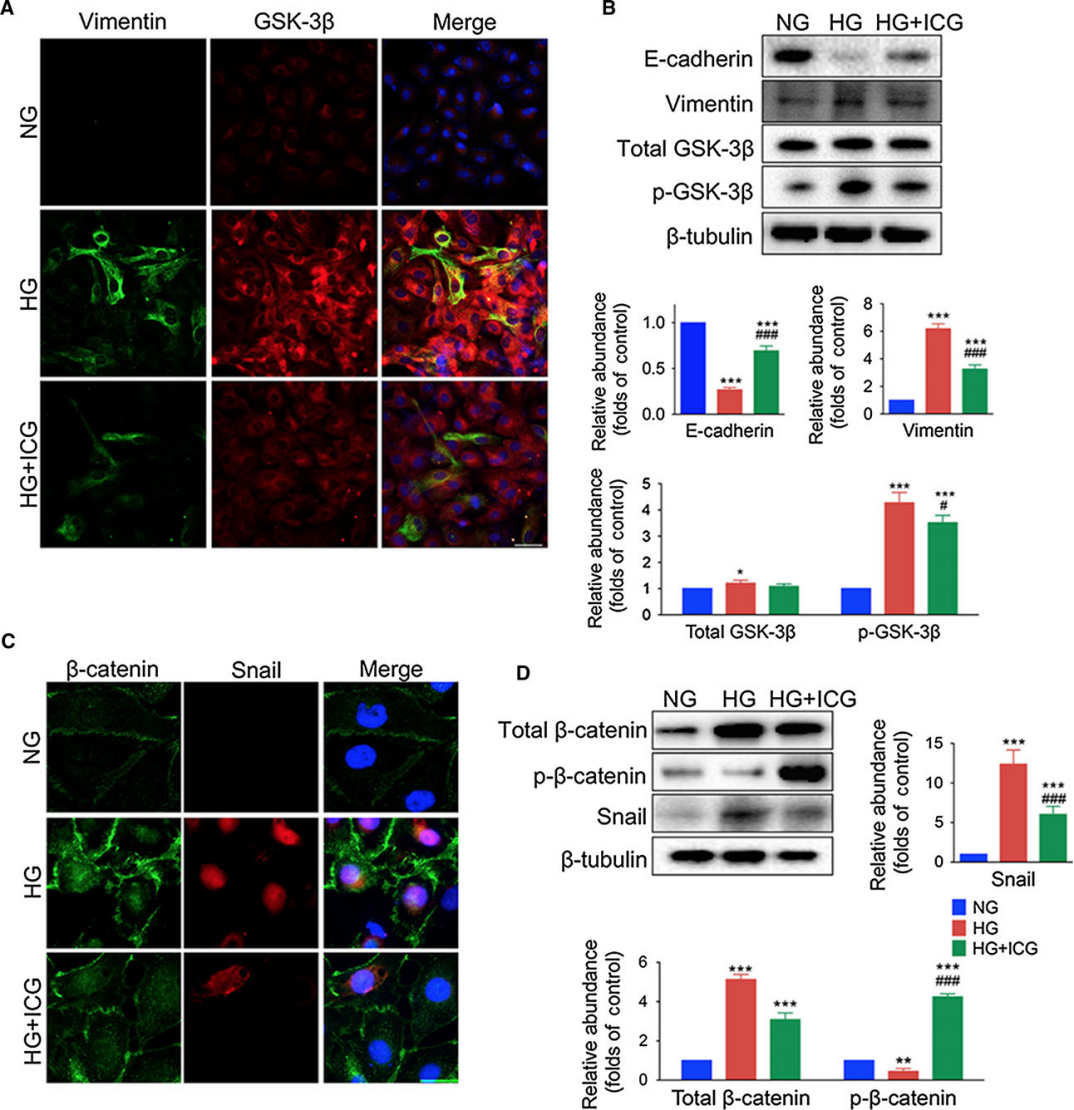
ICG‐001 reverses the expression of Vimentin and E‐cadherin, reactivates GSK‐3β, and inhibits Snail expression in mPECs. (A) Images of mPECs stained with antibodies against Vimentin (green) and GSK‐3β (red), and nuclei stained with Hoechst (blue). mPECs were exposed to normal glucose (NG), high glucose (HG, 4.25% d‐glucose), or high glucose with ICG‐001 (HG+ICG, 10 μm) for 48 h (original magnification, ×200; scale bar, 100 μm). (B) Upper panel, western blots of E‐cadherin, Vimentin, p‐GSK‐3β, total GSK‐3β, and β‐tubulin in mPECs. Lower panel, statistics for expression levels of E‐cadherin, Vimentin, p‐GSK‐3β, and total GSK‐3β normalized to β‐tubulin (mean ± SD, *n* = 4, ***P < 0.001 and *P < 0.05 *vs* NG group, ^###^ *P* < 0.001 and ^#^ *P* < 0.05 *vs* HG group). (C) Images of mPECs stained with antibodies against β‐catenin (green) and Snail (red), and nuclei stained with Hoechst (blue) (original magnification, ×650; scale bar, 40 μm). (D) Upper left panel, western blots of phosphorylated β‐catenin (p‐β‐catenin), total β‐catenin, and Snail in mPECs. Other panels, expression levels of p‐β‐catenin, total β‐catenin and Snail normalized to β‐tubulin (mean ± SD, *n* = 4, ***P < 0.001 and **P < 0.01 *vs* NG group, ^###^ *P* < 0.001 *vs* HG group).

Similar to the experiments on the effect of PDF on the peritoneum, we examined the localization of GSK, β‐catenin, and Snail by immunofluorescence and western blotting analysis of mPECs. We found that both total and phosphorylated GSK‐3β were up‐regulated by HG incubation, while p‐GSK‐3β demonstrated much larger significance (Fig. 4A,B). Parallel results showed that membrane‐bound, cytosolic, and nuclear translocation of β‐catenin and Snail were induced by HG incubation (Fig. 4C). The total amount of β‐catenin, as well as Snail expression measured by western blot, were markedly higher in the PDF group than the control group (Fig. 4D). We also compared mPECs incubated with NG and 4.25% l‐glucose, and found no significant difference in β‐catenin level, illustrating that osmotic pressure variations had little effect on the activation of β‐catenin expression (Fig. S1).

### β‐catenin participates in peritoneal fibrosis and cell proliferation

To assess whether activation of the β‐catenin pathway contributes to the onset of peritoneal fibrosis, we introduced the specific inhibitor ICG‐001 along with PDF injections. Daily ICG‐001 treatment significantly reduced the thickness of the submesothelial zone, as well as the number of BrdU‐positive cells in the peritoneum (Fig. 1) compared with the PDF group.

### β‐catenin participates in the EMT in peritoneal fibrosis

ICG‐001 reversed the decline of E‐cadherin and the increase in Vimentin expression in the peritoneum induced by exposure to PDF (Fig. 2). Immunofluorescence showed high levels of colocalization of β‐catenin and Snail in the nucleus of peritoneal cells from PDF‐treated mice and this was reduced by ICG treatment (Fig. 3A).

Immunofluorescence showed that ICG‐001 decreased the number of HG‐induced Vimentin‐positive cells (Fig 4A), and the colocalization of up‐regulated β‐catenin and Snail in the nucleus (Fig. 4C). The Vimentin, p‐GSK‐3β, total β‐catenin, and Snail levels measured by western blot were markedly higher in the HG group than in controls and were significantly lower in the HG+ICG group, while the E‐cadherin and phosphorylated β‐catenin levels were markedly lower in the HG group and higher in the HG+ICG than in controls, no significant difference was observed of total GSK between HG+ICG and control group (Fig. 4B–D). Lentivirus‐mediated shRNA knockdown of β‐catenin affected the morphology of mPECs (Fig. S2) and the EMT transitioning of mPECs induced by HG (Fig. S3). NG increased phosphorylated GSK‐3β in β‐catenin knockdown mPECs compared with Scramble cells.

## Discussion

Peritoneal fibrosis is one of the most common complications of long‐term PD 11. Exposure to PDF causes structural and functional deterioration of the peritoneal membrane, leading to ultrafiltration failure and the eventual withdrawal of PD treatment 12. Variation in the osmotic pressure had little effect on the activation of β‐catenin signaling process and downstream peritoneal fibrosis.

We used the marker proteins E‐cadherin and Vimentin to indicate the EMT process. The increased thickness and cell proliferation in the parietal peritoneum were accompanied by a loss of E‐cadherin and an increase in Vimentin expression, demonstrating that the EMT not only occurred in resting cells but also in the processes of proliferation and differentiation.

β‐catenin is one of the cytoskeletal proteins on the cell membrane, and establishes adherent junctions with E‐cadherin and α‐catenin on the cell membrane. The E‐cadherin‐β‐catenin complex is associated with actin filaments, thus forming a dynamic link with the actin cytoskeleton 13. This is also necessary for the creation and maintenance of epithelial cell layers and barriers. The β‐catenin‐α‐catenin complex physically bridges E‐cadherins with the actin cytoskeleton, and is regulated through phosphorylation and endocytosis. Previously, we treated mPECs with siRNA targeting β‐catenin mRNA, and the cells had disturbed shapes, had a low proliferation rate, and were unable to form cell–cell junctions compared with mPECs transfected with scrambled shRNA (Fig. S2), so we chose to inhibit β‐catenin signaling pharmacologically using ICG‐001. Otherwise, β‐catenin knockdown induced an increase in phosphorylated GSK‐3β. Furthermore, HG‐induced phosphorylated GSK‐3β increased in both Scr‐shRNA and β‐catenin‐shRNA cells (Fig. S3). To our understanding, the relative lower increase in phosphorylated GSK‐3β in ShRNA mPECs by HG could explain an inhibitory effect of EMT transition. The β‐catenin level was significantly reduced in ShRNA cells both incubated by NG and HG solution because of knockdown of β‐catenin genes, which was easy to understand. Our results showed a narrow distribution of β‐catenin at the cell–cell interface in the NG group. After 48 h of incubation with HG, a zig‐zag distribution of β‐catenin appeared on the cell surface, indicating structural distortion of the arrangement of epithelial cell–cell adhesion. ICG‐001 decreased the deposition and deformation of β‐catenin expression on the surface.

It is well established that β‐catenin participates in regulating the EMT during organ development and tumor metastasis, as well as wound‐healing and organ fibrosis 14. GSK‐3β is a serine–threonine protein kinase 15, and plays a key role as a negative regulator of β‐catenin, and its phosphorylation alleviates the degradation of β‐catenin *via* ubiquitination, assisted by the GSK‐3β‐axin‐APC complex 16, 17. In our study, up‐regulated phosphorylation of GSK‐3β caused by PDF treatment was followed by augmentation of β‐catenin accumulation in both the cytosol and nucleus. This indicates that inhibition of GSK‐3β is involved in the upstream signal of PDF‐induced β‐catenin activation. The increased intracellular β‐catenin induces migration into the nucleus to form β‐catenin/TCF/LEF complexes followed by transcription of target genes such as fibronectin, Twist, and Snail, which are key nuclear effectors in the EMT process associated with damage and repair 18, 19. Snail is one of the nuclear effectors of β‐catenin, and also plays a positive‐feedback role in the EMT in association with β‐catenin 20. We found that elevation of nuclear β‐catenin was also positively associated with Snail expression. It is worth noting that a slight distinction of total GSK‐3β was acquired from *in vivo* and *in vitro* experiments. A stepwise ascension was observed among the peritoneal tissues from Control, PDF, and PDF+ICG groups, while only a slight increase in total GSK‐3β was induced by HG in the cultured mPECs, and the muscular tissue of the peritoneum involved in the western blotting might contribute to the difference. ICG‐001 is a well‐known selective inhibitor of the β‐catenin pathway 21, which disrupts the interaction of β‐catenin with TCF by binding to the transcriptional coactivator cyclic AMP response element‐binding protein 22. Its effect against the EMT has been demonstrated in colon adenocarcinoma cells and acute lymphoblastic leukemia cells 23. ICG‐001 also had a significant effect on the change in phosphorylation levels of β‐catenin and GSK‐3β in mPECs, reflecting the feedback effect of β‐catenin target genes on GSK‐3β expression and inhibition 19.

In summary, activation of the β‐catenin pathway participates in the EMT process in peritoneal epithelial cells and is one of the underlying mechanisms of this pathological change, so a strategy of blocking the EMT in the peritoneum by blocking the β‐catenin pathway might be used to prevent peritoneal fibrosis.

## Materials and methods

### Animals and materials

The experimental protocol was approved by the Ethics Committee for the Use of Experimental Animals in Zhejiang University (No. 2016‐279) and was carried out in accordance with the National Institutes of Health Guide for the Care and Use of Laboratory Animals (NIH Publications No. 80‐23). Two‐month‐old male C57BL/6 mice were provided by the Experimental Animal Center in Zhejiang Academy of Medical Sciences (Hangzhou, Zhejiang, China). Mice were maintained on standard rodent chow and had free access to food and water under a 12/12 h light/dark cycle, and BrdU was from Sigma (St. Louis, MO, USA). PDF with 4.25% glucose was from Baxter (Chicago, IL, USA). ICG‐001 was from Tocris Bioscience (Ellisville, MO, USA). Antibodies against β‐catenin, E‐cadherin, and Vimentin were from Cell Signaling Technology (Danvers, MA, USA). Antibodies against Snail and GSK‐3β were from Abcam (Cambridge, MA, USA).

### PDF‐induced peritoneal fibrosis in mice

Fifteen C57BL/6 mice were randomly divided into three groups. The mice in the control group were given 10% of their body weight of saline by intraperitoneal injection per day. The PDF mice received 10% of their body weight of 4.25% glucose PDF, while the PDF+ICG mice received the same volume of PDF combined with ICG‐001 (5 mg·kg^−1^·day^−1^) 22. All mice were given BrdU (5 mg·kg^−1^, intraperitoneal) every 2 days. Mice were deeply anesthetized and sacrificed by cervical dislocation after 30 days, and peritoneal tissues were obtained for histopathology.

### mPEC culture and treatment

Mice peritoneal epithelial cells were collected by needle aspiration 5 min after intraperitoneal injection of 2 mL of 0.25% trypsin‐EDTA, and cultured in Dulbecco's modified Eagle's medium (DMEM/F12) with 10% FBS (all from Thermo Fisher, Waltham, MA, USA) in a 5% CO_2_ incubator at 37 °C. HG was induced with 4.25% d‐glucose, and high osmotic pressure was induced with 4.25% l‐glucose. Cells were cultured with or without ICG‐001 (10 μm) for 48 h and then harvested for immunofluorescence staining and protein expression measurement.

### Morphology, immunohistochemistry, and immunofluorescence analysis

Peritoneal tissues fixed in 4% paraformaldehyde were dehydrated in ethanol and cleared in xylene, then embedded in paraffin and cut into 4‐μm sections. To evaluate peritoneal fibrosis, sections were stained with Masson's trichrome, and 10 images at high magnification (×400) were assessed for each animal; the average value for five sites was calculated to evaluate the thickness of peritoneal tissue.

Antibodies against BrdU, E‐cadherin, and Vimentin were used to label the paraffin sections, and antibodies against β‐catenin and Snail were used to label the frozen sections. Briefly, sections were blocked with 5% goat serum for 1 h, incubated with primary antibodies overnight at 4 °C, and labeled with the appropriate secondary antibodies.

Mice peritoneal epithelial cells were fixed in 4% paraformaldehyde for 15 min, permeabilized with 0.1% Triton X‐100 for 15 min, and blocked in 10% goat serum in PBS for 1 h at room temperature. Antibodies against Vimentin, GSK‐3β, β‐catenin, and Snail were used. Nuclei were counterstained with Hoechst (1 : 80 000; Thermo Fisher) for 5 min. Images were captured on a fluorescence microscope (Leica, Shanghai, China) and analyzed using imagej (NIH, Bethesda, MD, USA).

### Western blotting

Tissues and mPECs were homogenized in RIPA lysis buffer with protease inhibitor cocktail and phosphatase inhibitor cocktail (CST, Danvers, MA, USA). Supernatants were assayed by western blot. Briefly, proteins were separated on 10% SDS/PAGE and electro‐transferred to nitrocellulose membranes, which were which were then blocked in 5% nonfat milk and probed with primary antibodies against E‐cadherin, Vimentin, β‐catenin, p‐β‐catenin, p‐GSK‐3β, Snail, and β‐tubulin followed by specific secondary antibodies. The membranes were scanned with an infrared imaging system (Gel Doc XR system; Bio‐Rad, Hercules, CA, USA), and the software provided with the system was used to evaluate the band intensity.

### Statistical analysis

Values are mean ± SD, and were compared by two‐way ANOVA with the Bonferroni post‐test from more than two groups (graphpad prism 6.0, GraphPad Software, Inc., La Jolla, CA, USA). Two‐tailed Student's *t*‐test was applied (graphpad prism 6.0). *P* < 0.05 was considered to be statistically significant.

## Author contributions

SJ designed and performed the *in vitro* experiments. HD performed the *in vivo* experiments and wrote the manuscript. PY and WJ performed the histochemistry experiments and the statistical analysis. RW, LP, JZ, XC, and JZ contributed to the cell culture and histochemistry experiments. Experiments were performed under the supervision of XZ and JS.

## Supporting information


**Fig. S1.** The effect of osmotic pressure change on β‐catenin in mPECs.

**Fig. S2.** Lentivirus‐mediated shRNA knockdown of β‐catenin affects the morphology of mPECs.

**Fig. S3.** Lentivirus‐mediated shRNA knockdown of β‐catenin inhibits EMT of mPECs induced by high glucose.
