# Supplementary figures and images for: Beta‐catenin participates in dialysate‐induced peritoneal fibrosis via enhanced peritoneal cell epithelial‐to‐mesenchymal transition
Source: FEBS Open Bio. 2017 Jan 19;7(2):265–73. doi: 10.1002/2211-5463.12182 (PMC5292666; doi:10.1002/2211-5463.12182)

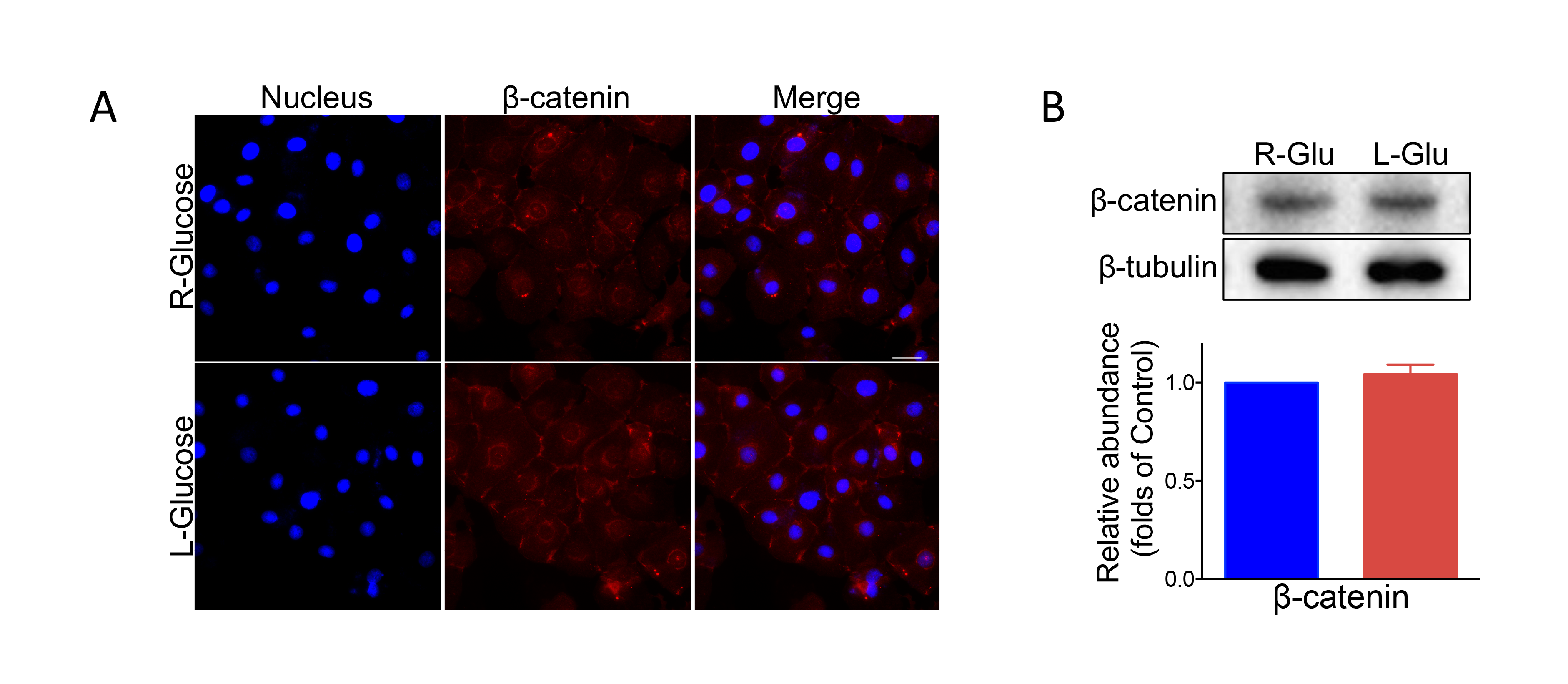

Supplement: Supplementary file 1 — Fig. S1. The effect of osmotic pressure change on β‐catenin in mPECs. [file FEB4-7-265-s001.tif]

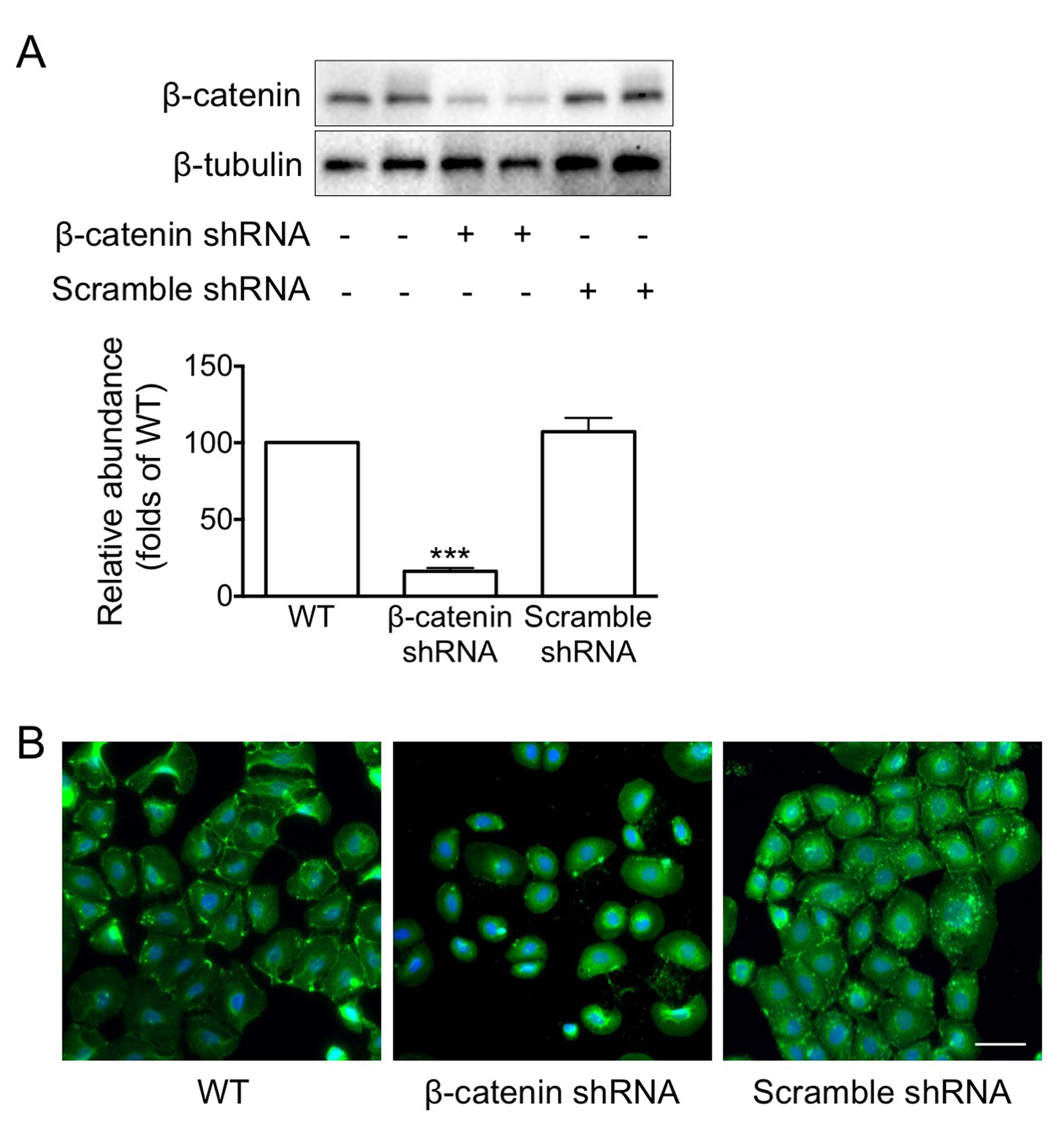

Supplement: Supplementary file 2 — Fig. S2. Lentivirus‐mediated shRNA knockdown of β‐catenin affects the morphology of mPECs. [file FEB4-7-265-s002.tif]

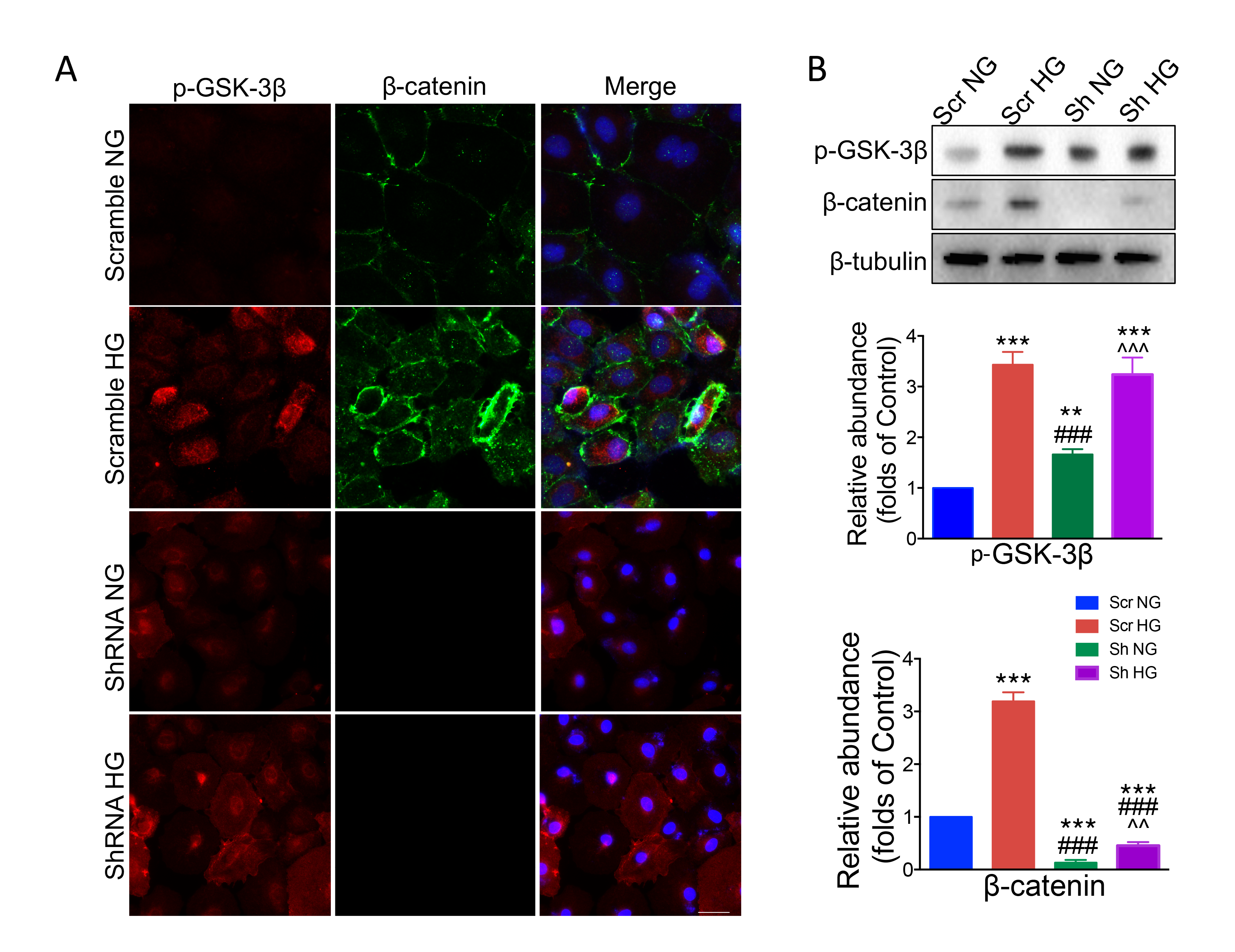

Supplement: Supplementary file 3 — Fig. S3. Lentivirus‐mediated shRNA knockdown of β‐catenin inhibits EMT of mPECs induced by high glucose. [file FEB4-7-265-s003.tif]
